# Unsaturated Fatty Acids Affect Quorum Sensing Communication System and Inhibit Motility and Biofilm Formation of *Acinetobacter baumannii*

**DOI:** 10.3390/ijms19010214

**Published:** 2018-01-10

**Authors:** Marion Nicol, Stéphane Alexandre, Jean-Baptiste Luizet, Malena Skogman, Thierry Jouenne, Suzana P. Salcedo, Emmanuelle Dé

**Affiliations:** 1 Normandie University, Unirouen, 76000 Rouen, France; marion.nicol@etu.univ-rouen.fr (M.N.); stephane.alexandre@univ-rouen.fr (S.A.); thierry.jouenne@univ-rouen.fr (T.J.); 2 CNRS, UMR 6270, Polymers, Biopolymers, Surfaces Laboratory, F-76821 Mont-Saint-Aignan, France; 3 Laboratory of Molecular Microbiology and Structural Biochemistry, University of Lyon, Centre National de la Recherche Scientifique, F-69367 Lyon, France; jean-baptiste.luizet@ibcp.fr (J.-B.L.); suzana.salcedo@ibcp.fr (S.P.S.); 4 Department of Pharmaceutical Biosciences, Faculty of Pharmacy, University of Helsinki, Viikinkaari 5E, FI-00014 Helsinki, Finland; malena.skogman@helsinki.fi

**Keywords:** palmitoleic acid, myristoleic acid, biofilm, pellicle, quorum sensing

## Abstract

The increasing threat of *Acinetobacter baumannii* as a nosocomial pathogen is mainly due to the occurrence of multidrug-resistant strains that are associated with the real problem of its eradication from hospital wards. The particular ability of this pathogen to form biofilms contributes to its persistence, increases antibiotic resistance, and promotes persistent/device-related infections. We previously demonstrated that virstatin, which is a small organic compound known to decrease virulence of *Vibrio cholera* via an inhibition of T4-pili expression, displayed very promising activity to prevent *A. baumannii* biofilm development. Here, we examined the antibiofilm activity of mono-unsaturated chain fatty acids, palmitoleic (PoA), and myristoleic (MoA) acids, presenting similar action on *V. cholerae* virulence. We demonstrated that PoA and MoA (at 0.02 mg/mL) were able to decrease *A. baumannii* ATCC 17978 biofilm formation up to 38% and 24%, respectively, presented a biofilm dispersing effect and drastically reduced motility. We highlighted that these fatty acids decreased the expression of the regulator *abaR* from the LuxIR-type quorum sensing (QS) communication system AbaIR and consequently reduced the *N*-acyl-homoserine lactone production (AHL). This effect can be countered by addition of exogenous AHLs. Besides, fatty acids may have additional non-targeted effects, independent from QS. Atomic force microscopy experiments probed indeed that PoA and MoA could also act on the initial adhesion process in modifying the material interface properties. Evaluation of fatty acids effect on 22 clinical isolates showed a strain-dependent antibiofilm activity, which was not correlated to hydrophobicity or pellicle formation ability of the tested strains, and suggested a real diversity in cell-to-cell communication systems involved in *A. baumannii* biofilm formation.

## 1. Introduction

*Acinetobacter baumannii* is a bacterial pathogen causing nosocomial outbreaks worldwide and is responsible for many infections, such as pneumonia and bloodstream infections, especially in intensive cares units [1,2]. Due to its exceptional adaptability to detrimental environmental conditions, this bacterial species has rapidly emerged as a Multi-Drug Resistant (MDR), but also XDR (extensively-DR) and now, more and more often, as a PDR (Pan-DR) organism. This led the World Health Organization to classify *A. baumannii* among the “Critical” bacterial agents (priority 1), for which research and development of new and effective antibiotic treatments are urgently required. Besides, this pathogen is also problematic for its long-time survival in hospital settings owing to its great ability to survive desiccation [3] or treatment with disinfectants [4]. This persistence is mostly linked to its capacity to form biofilms [5,6]. Virstatin is known to inhibit expression of cholera toxin (encoding by *ctx* genes) and toxin co-regulated pilus (a type IV pilus, T4P, encoding by *tcp* genes), two major virulence factors of *Vibrio cholerae*. We previously demonstrated that this small organic molecule prevents *A. baumannii* biofilm production possibly via inhibition of pili biosynthesis [7,8,9]. Virstatin antibiofilm activity was recently confirmed on *Acinetobacter nosocomialis* [10], and could be due to an inhibition of the Quorum-sensing (QS) system. QS is a communication system that orchestrates bacterial behaviors within a microenvironment to promote community establishment by the regulation of specific genes. In most gram-negative bacteria, signal molecules, called acyl-homoserine lactones (AHLs), are diffusible autoinducers that are characterized by a length variable acyl-chain coupled with a homoserine lactone ring [11]. In *A. baumannii*, different type of AHLs have been described [12], the most commonly described ones are long chain AHLs with C_10_ or C_12_ acyl chains [13,14,15]. When considering its crucial involvement in biofilm development, QS is an interesting target for the development of antibiofilm strategies that can act either by inhibiting the signal molecule synthesis, or by degrading or quenching this signal in the external environment [12]. Some mono-unsaturated fatty acids (UFAs) as palmitoleic (*cis*-9-hexadecenoïc, C16:1Δ9, PoA) and myristoleic (*cis*-9-tetradecenoïc, C14:1Δ9, MoA) acids were shown to inhibit *tcp* genes expression in *V. cholerae* [16,17]. These molecules prevent the interaction between their transcriptional regulator ToxT and the DNA [18]. Bactericidal activity of UFAs, in particular against cutaneous pathogens, has already been described [19,20,21]. Besides, UFAs can also affect virulence factor expression, initial adhesion, or motility [20]. In this study, we evaluated the efficacy of unsaturated fatty acids, PoA and MoA, as antibiofilm compounds and investigated their effect on *A. baumannii* QS system.

## 2. Results and Discussion

### 2.1. Effect of UFAs on A. baumannii ATCC 17978 Biofilm Growth and Motility

**Figure 1:**
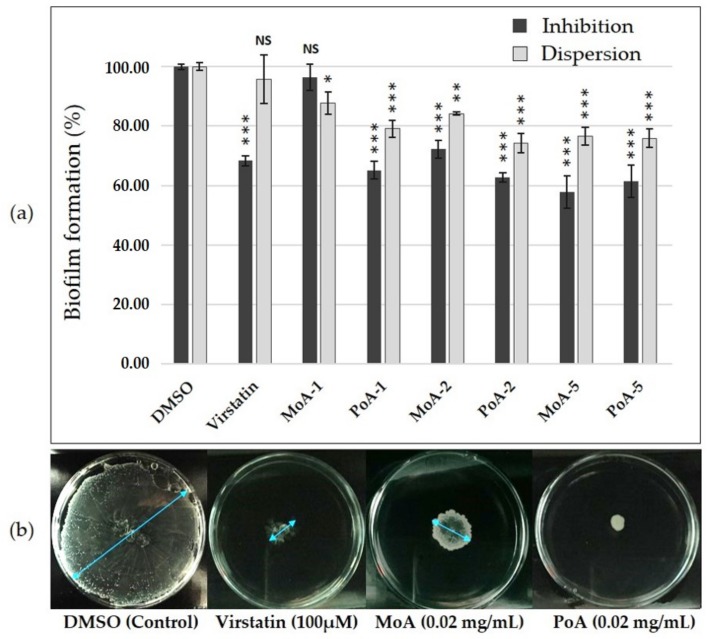
UFAs activity on *A. baumannii* ATCC 17978 motility and biofilm formation. (**a**) Inhibition and dispersion of biofilms quantified by crystal violet staining method. 24 h-biofilms were treated with or without virstatin (100 µM), palmitoleic acid (PoA) or myristoleic acid (MoA) at different concentrations (0.01 (UFA-1), 0.02 (UFA-2) or 0.05 (UFA-5) mg/mL) and DMSO as control; (**b**) Activity on motility. Blue arrows measure the diameter of surface motility. Results are presented as (mean ± standard error of mean). “***” for *p* < 0.0001, “**” for *p* < 0.01, “*” for *p* < 0.05 and “NS” for non-significant difference.

Activity of PoA and MoA was preliminary tested on *A. baumannii* ATCC 17978 reference strain forming both a biofilm at the solid-liquid interface and a pellicle. In the planktonic growth mode, MICs of 4 mg/mL were obtained for each UFA. To investigate the antibiofilm activity of these compounds, we used sub-inhibitory concentrations at least 100-fold lower than the MICs, i.e., 0.01, 0.02 and 0.05 mg/mL, concentrations in agreement with those used to decrease production of T4P in *V. cholerae* [17]. At these concentrations, fatty acids did not modify bacterial growth (Figure S1). The biofilm formation inhibition by fatty acids is clearly depicted by the Figure 1a. Addition of PoA reduced significantly the biofilm formation at the three tested concentrations (up to 37% and 39% reduction at 0.02 and 0.05 mg/mL, respectively), whereas MoA exhibited a significant activity only at 0.02 and 0.05 mg/mL (decrease of 28% and 42% respectively). These results showed that UFAs display a biofilm inhibition activity that is similar to that of virstatin, for which the decrease reached 32%, MoA being however less active than PoA at lower concentrations. Biofilm dispersion activity of UFAs was investigated on 24 h-static biofilms. Incubation of biofilms with MoA or PoA for anadditional 24 h demonstrated that these UFA displayed significant dispersive activity as compared to virstatin (decrease of 24% for MoA and PoA at 0.05 mg/mL, Figure 1a). Finally, *A. baumannii* surface motility was tested on semi-solid medium plate (0.3% agar) with or without UFAs (Figure 1b at 0.02 mg/mL and Table S1). PoA impeded motility when added at 0.02 and 0.05 mg/mL and MoA also significantly decreased the motility up to 73% at 0.05 mg/mL.

These results showed that UFAs prevent significantly motility and biofilm formation on *A. baumannii* ATCC 17978 at sub-inhibitory concentrations (0.01, 0.02 and 0.05 mg/mL) with a better activity of PoA (C16:1Δ9) than MoA (C14:1Δ9). This is in agreement with the observation that, in *V. cholera*, UFAs activity, reducing the *tcp* gene expression, was improved by an increase of the length chain and was also related to the presence of unsaturated bond and to the conformation (*cis*/*trans*) of the molecule [16,17]. Moreover, unlike virstatin, PoA and MoA were shown to significantly disperse 24 h-biofilms. Some Gram-bacteria produced *cis*-UFAs, also called Diffusible Signaling Factors (DSF). These QS signals have been shown to be involved in cell-to-cell communication and could regulate biofilm lifestyle [22]. For example, the UFA *cis*-2-decenoic acid (CDA) from the DSF family was shown to be an autoinducer of the biofilm dispersion in *P. aeruginosa* and in many others species [23,24,25,26]. PoA and MoA might possess similar activity in *A. baumannii* biofilms. It was already shown that the addition of an exogenous DSF, i.e., *cis*-2-dodecenoic (BDSF), inhibits *P. aeruginosa* biofilm formation by interfering with the production of AHL [27]. This prompted us to further examine the impact of UFAs on *A. baumannii* QS.

### 2.2. Impact of UFAs on Quorum Sensing

**Figure 2:**
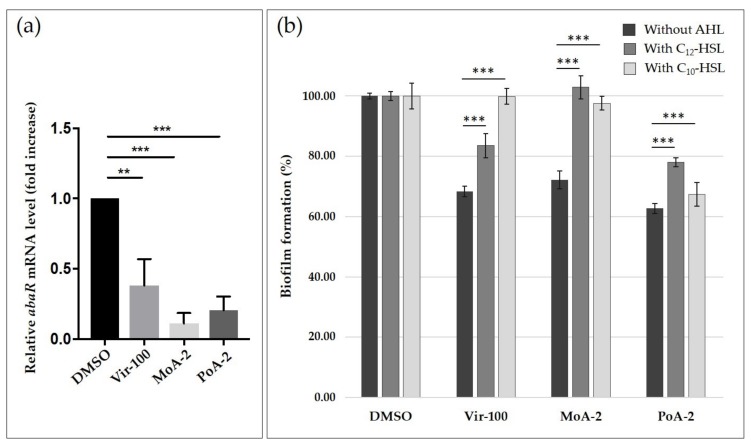
UFAs activity on *A. baumannii* ATCC 17978 quorum sensing system. (**a**) *abaR* gene expression quantified by real time PCR of the total RNA isolated from bacteria grown in the presence of virstatin (100 µM, Vir-100), PoA or MoA at 0.02 mg/mL (PoA-2 and MoA-2) relative to that of the bacteria grown in DMSO alone; (**b**) UFAs activity on biofilm formation in presence of AHLs (500 nM) quantified by crystal violet staining method. 24 h-biofilm formation with or without virstatin (100 µM), PoA or MoA at 0.02 mg/mL and DMSO as control. Results are presented as (mean ± standard error of mean). “***” for *p* < 0.0001, “**” for *p* < 0.01 and “NS” for non-significant difference.

It was previously shown that virstatin could interfere with QS system of *A. nosocomialis*, by decreasing the expression of the *anoR* regulator of the LuxI/R-type AnoIR system [10]. This decrease of *anoR* expression, in reducing the activation of *anoI*, gene that codes for the autoinducer synthase, could decrease AHLs production. In order to determine if virstatin or UFAs could also impact the QS system in *A. baumannii* ATCC 17978, we examined the expression of the regulator *abaR* gene of the AbaIR system (homologue of the *anoR* gene in *A. nosocomialis*). We found that virstatin, MoA or PoA significantly decreased *abaR* expression suggesting these UFAs can interfere with the AbaIR QS system of *A. baumannii* (Figure 2a and Table S1).

To confirm the impact of these compounds on the production of AHLs, we first performed cross-streaking of *A. tumefaciens* and *C. violaceum* biosensors against *A. baumannii* ATCC 17978 to determine the type of AHLs, i.e., short or/and long-type, which are produced by this strain [28]. In agreement with previous studies [13,14], we only detected the production of long-AHLs by *A. baumannii* ATCC 17978 (Figure S2). To further examine the activity of UFAs and virstatin on AHLs production, we evaluated the activity of these compounds on the *A. baumannii* biofilm formation in presence of 500 nM of the main AHLs already described, i.e., OH-C_12_-HSL or N-C_10_-HSL [13,14,15] (Figure 2b). If OH-C_12_-HSL addition completely restored the biofilm formation when *A. baumannii* was treated by MoA, it only partially counteracted the activity of virstatin and PoA (recovery of 54 and 38% of the phenotype, respectively). The addition of N-C_10_-HSL also totally inhibited the effect of MoA as well as the effect of virstatin (98% and 100% of recovery, respectively), whereas no significant activity of N-C_10_-HSL was shown on biofilms treated with PoA (only 4% of recovery, Figure 2b). Addition of AHLs on motility plates did not restore the motility of *A. baumannii* ATCC 17978 abolished by virstatin or UFAs. Finally, we tested virstatin and UFAs via the QS screening platform developed by Skogman et al. [29]. Neither virstatin nor UFAs (up to 400 µM) could be characterized as quorum quencher or quorum inhibitor of short AHLs production.

In line with the previous data obtained on *A. nosocomialis* [10], these overall results indicate that virstatin, but also UFAs, could prevent biofilm formation via an inhibition of *abaR* gene expression. The consequent *abaI* autoinducer synthase gene repression could thus lead to an inhibition of the long-AHLs production. It has been shown that a deletion of the *abaI* in *A. baumannii* M2 displayed a 40% reduction of biofilm formation [13], a decrease that is similar to the one induced by virstatin or UFAs. For PoA, the partial recovery of biofilm formation after addition of C_10_- or C_12_-HSL suggests that this UFA alters the AHLs production, but could also act on another QS communication system not yet characterized.

### 2.3. UFAs Affect Biofilm Architecture

**Figure 3:**
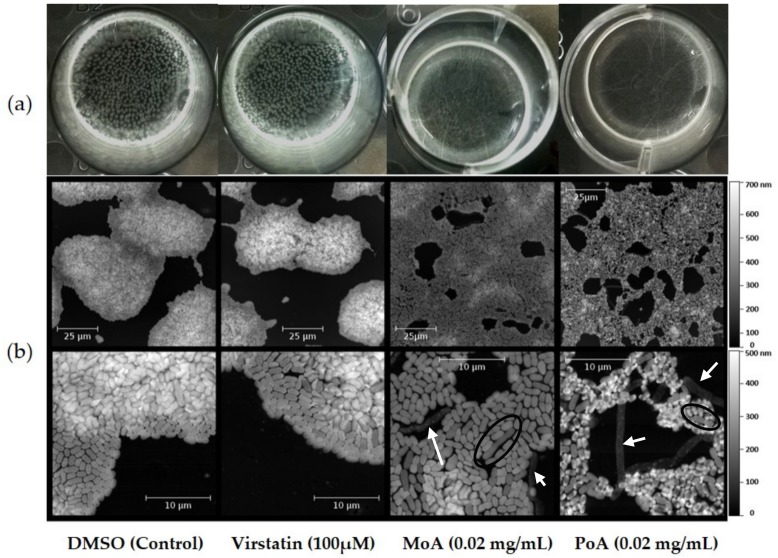
UFAs activity on air-liquid biofilm organization. Pellicles were observed after growth with or without virstatin, PoA or MoA. (**a**) Visual aspect of pellicle surface after 24 h treatment (**b**) Atomic Force Microscopy (AFM) images of pellicle water-facing sides after 6 h treatment. From left side to right side: DMSO control, pellicle formation with 100 µM virstatin, with 0.02 mg/mL MoA and with 0.02 mg/mL PoA. Elongated cells with a normal thickness are black encircled and lysed elongated cells are pointed out with white arrows.

We next investigated pellicles formed by ATCC 17978 after treatment with either virstatin or UFAs. The pellicle formed by the ATCC 17978 strain showed aggregates on the air-facing side (the “ball-shaped” morphogroup according to [30]) in control growth or in the presence of virstatin. After 24 h of UFA treatment, these aggregates disappeared (Figure 3a). Pellicles were further characterized by AFM. After 6 h of growth, control and virstatin-treated pellicles exhibited a similar macroscopic aspect with merging microcolonies characterized by a diameter of about 50–100 µm and an average thickness of (360–460 ± 40) nm (Figure 3b). However, with UFAs, tridimensional architecture was abolished, leading to a monolayer structure with an average thickness of (230 ± 40) nm and (150 ± 40) nm with PoA and MoA, respectively. This monolayer cellular organization appeared to be less cohesive after PoA treatment than after the MoA one. We also observed elongated cells inside the pellicle formed with and without UFAs (up to 20 µm of length versus 1 µm average for normal cells, Figure 3b). When these elongated cells were inside the biofilm, they exhibited the same thickness than normal cells (black circles in Figure 3b). However, in the presence of PoA or MoA, a large part of these atypical cells seem to be lysed and their membranes expelled from the community (white arrows in Figure 3b). UFC counts in the biofilms after 24 h with or without the presence of UFAs or virstatin were similar (Figure S1).

These observations suggest a specific action of UFAs during the initial steps of biofilm formation, i.e., on the cell adhesion. Indeed, due to their amphiphilic nature, UFAs go spontaneously at the air-liquid interface where they could locally accumulate and lead to a first antibiofilm action. In agreement with this hypothesis, it was previously shown that oleic acid (C18:1Δ^9^, OA) inhibited the primary adhesion step of *S. aureus* on polystyrene surfaces [31]. We also observed that the addition of UFAs in MH medium significantly reduced the air/liquid surface tension. It has been already demonstrated that this type of interface alteration, due to addition of biosurfactants, for an example, significantly reduced pellicle formation or induced biofilm dispersion [32,33]. Finally, the observation of the air-liquid interface by Brewster Angle Microscopy [30] demonstrates that if DMSO did not influence the organization of the monolayer formed by the growth medium molecules, in the presence of UFAs, the fluidity of this monolayer drastically increased. The influence of this parameter on the pellicle formation is difficult to evaluate, but one might suggest that the irreversible adhesion step, preluding microcolony formation during biofilm development, might be more difficult to achieve for bacteria on fluid surface. Taken together, these overall results suggest that, besides its action on QS system, the antibiofilm activity of UFAs could also be due, at least in initial steps of biofilm formation, to several modifications of the interfaces on which the biofilm settles.

### 2.4. UFAs Effect on Biofilm Formation and Mobility of Clinical Isolates

To evaluate more broadly the UFAs activity on *A. baumannii* biofilms, we tested MoA and PoA on 22 clinic isolates from different origins [34]. We quantified the biofilm formation of these strains grown with or without UFAs at 0.02 mg/mL. PoA decreased significantly the biofilm formation in 13 of strains with a maximum reduction of 44% (Figure S3). With MoA, eight strains exhibited a significant reduction of their biofilm formation ability. As observed with the ATCC 17978 strain, PoA displayed a better antibiofilm activity than MoA, but no relationship between the antibiofilm activity of virstatin and the one of UFAs could be emphasized [7]. UFAs antibiofilm activity seems not to be correlated to the pellicle formation ability or hydrophobicity of the strain (Figure S3). For some strains, UFAs addition slightly promoted the biofilm formation. Cross-streaking of *A. tumefaciens* biosensor against these isolates confirmed the efficiency of the AHL QS system by long AHL production. Several hypotheses can be proposed to explain this increase. In *S. aureus*, it was shown that OA could promote biofilm formation, probably by interaction between positive charges of adhesion factors and negative charges of UFAs [31,35]. Expression of adhesion factors in *A. baumannii* was shown to be strain-dependent [36] and might explain this increasing effect. One can notice also that the quorum quencher enzyme MomL displayed on *A. baumannii* biofilms a similar activity as UFAs, with a maximum 42% decrease of the biomass formation and a strain-dependence of its activity [37], suggesting that other cell-to-cell communication systems or factors could be recruited during *A. baumannii* biofilm formation. In *V. cholerae*, UFAs (PoA and OA) were shown to interact and prevent the DNA interaction of the AraC-type regulator, ToxT [17]. Additional interaction of UFAs with such regulators in clinical isolates may also explain an increased biofilm formation. This demonstrates that the UFAs activity is not limited to an activity an *abaR* gene.

## 3. Materials and Methods

### 3.1. Bacterial Strains and MICs Determination

To evaluate fatty acid activity, we used two reference strains of *A. baumannii* (i.e., ATCC 17978 and ATCC 19606) and a panel of 22 *A. baumannii* clinical isolates previously described [7,34]. Fatty acids, *cis*-9-hexadecenoic acid (C16:1Δ9, palmitoleic acid, PoA, Sigma Aldrich, Lyon, France) and *cis*-9-tetradecenoic acid (C14:1Δ9, myristoleic acid, MoA, Sigma Aldrich, France) and virstatin (4-[*N*-(1,8-naphthalimide)]-n-butyric acid, Bachem, Bubendorf, Germany) were solubilized in dimethyl sulfoxide (DMSO, Sigma, St. Louis, MO, USA). Determination of UFAs minimal inhibitory concentrations (MIC) were performed by the microdilution method, as previously described by [7].

### 3.2. UFAs Activities on Biofilm Formation and Motility of A. baumannii Strains

*A. baumannii* biofilms were grown on 24-well plates in Mueller Hinton broth (MHB, Difco, Sparks, NV, USA) at 37 °C as previously described [7]. To test their inhibition activity on *A. baumannii* biofilms, fatty acids were introduced at final concentration of 0.02 mg/mL (as well as 0.01 and 0.05 mg/mL for ATCC 17978 strain), using DMSO as negative control and 100 µM virstatin as positive control [7,17]. In case of strains forming pellicle in addition to biofilms on plate walls, the sub-phase was gently removed allowing the pellicle to stick onto the plate walls, then the overall remaining biomass (i.e., biofilm on solid surface and pellicle) was quantified, as described by [38]. Biofilm dispersion activity of UFAs was investigated only on 24 h-biofilms formed by ATCC 17978 strain in MHB. Twenty four-hour biofilms were incubated in fresh medium for an additional 24 h in the presence of fatty acids (at 0.01, 0.02, or 0.05 mg/mL) using DMSO or virstatin 100 µM as controls. Surface motility of *A. baumannii* strains was investigated in 0.3% Luria Bertani agar (LB; Difco) Petri dishes supplemented or not with UFAs and DMSO as control and incubated overnight at 37 °C [39]. All of the experiments were performed at least in triplicate. Data were statically analyzed using Prism Graph Pad 5 with a *t*-test to determine a significant effect of UFAs.

### 3.3. Effect of Virstatin and UFAs on Quorum Sensing

The *N*-acyl-homoserine lactone (AHL) production in *A. baumannii* ATCC 17978 or *A. baumannii* clinical isolates was determined, as described by [28] with *P. aeruginosa* PAO1 and *E. coli* ATCC 10536 as positive and negative controls, respectively. To investigate the potential quorum-quenching activity of UFAs and virstatin on short-HSLs, we used the screening platform described by [29]. The effect of the addition of long-HSLs, i.e., *N*-(3-hydroxydodecanoyl)-dl-homoserine lactone (OH-C_12_-HSL, Sigma Aldrich) and *N*-decanoyl-dl-homoserine lactone (N-C_10_-HSL, Sigma Aldrich) solubilized in DMSO, on biofilms pretreated by virstatin and UFAs was investigated. Five hundred nM of each HSL were added concomitantly to 0.02 mg/mL of UFAs and 100 µM virstatin. DMSO enriched with HSL (500 nM) was used as control. Biofilm formation was quantified and the results significance was analyzed, as described in the previous section. The effect of virstatin and UFAs on *abaR* gene expression using q-RT-PCR was also investigated. *A. baumannii* was grown overnight in MH medium and diluted to OD600 of 0.01 and incubated for 24 h under agitation at 37 °C with DMSO (negative control), virstatin (100 μM), MoA (0.02 mg/mL), or PoA (0.02 mg/mL). Total RNAs were extracted using RNeasy-Mini kit (Qiagen, Valencia, CA, USA), followed by a supplementary DNase treatment (Ambion, Carlsbad, CA, USA), and a verification of absence of contaminating DNA by PCR. RNAs were reverse-transcribed using QuantiTect Reverse Transcription Kit (Qiagen). Real-time PCR was performed using Brilliant III SYBR Green QPCR (Agilent Technologies, Santa Clara, CA, USA) with an AriaMx Real-Time PCR System (Agilent Technologies). Specific primers are listed in Table S1. The *rpoB* expression was used as an internal control for normalization and fold change calculated in relation to the DMSO control. Data were analyzed using Prism Graph Pad 5 with a one-way ANOVA (*n* = 4).

### 3.4. Impact of UFAs on A. baumannii Biofilm Morphology

Morphological changes of liquid-facing sides of *A. baumannii* ATCC 17978 pellicles were visualized by Atomic Force Microscopy (AFM) after 6h of growth with or without 0.02 mg/mL of UFAs or 100 µM virstatin, DMSO being used as control [7]. Modifications of air-water interfaces in presence of UFAs and virstatin, were determined either by Brewster’s angle microscopy [30] or by surface tension measurements using a Wilhelmy pressure sensor with a filter paper plate (R & K, Wiesbaden, Germany).

## 4. Conclusions

Involved in device-related infections and in persistence in hospital settings, the biofilm formation is a major cause of concern in the battle against *A. baumannii* and deserves the research of new therapeutic compounds. In this context, we previously demonstrated that virstatin, a factor decreasing T4P-pili expression in *V. cholera* [17], inhibits *A. baumannii* biofilm formation [7]. In this study, we demonstrated that MoA and PoA also decreasing T4P-pili expression in *V. cholera* [16,17], inhibit the biofilm formation and motility in *A. baumannii*. By decreasing the expression of the QS system regulator, *abaR*, these three compounds inhibit AHLs production that could be restored by AHL exogenous addition. However, UFAs, as hydrophobic compounds, also modify material interfaces, as shown here by the modification of the air-liquid interface, precluding initial steps of biofilm formation. Additional bacterial responses that are uncorrelated to QS communication system or sensing of surfaces might also be induced by the use of these compounds, like overexpression of genes involved in stress response, or the regulation of peptidoglycan biosynthesis highlighted in *S. aureus* under OA treatment [40], and merit further investigation.

## Supplementary Materials

Supplementary materials can be found at www.mdpi.com/1422-0067/19/1/214/s1.

## Abbreviations

AFM	Atomic force microscopy
AHL	*N*-acyl-homoserine lactone
ATCC	American type culture collection
DMSO	Dimethyl sulfoxide
HSL	Homoserine lactone
MIC	Minimal inhibitory concentration
MoA	Myristoleic acid
PoA	Palmitoleic acid
OA	Oleic acid
PCR	Polymerase chain reaction
QS	Quorum sensing
UFA	Unsaturated fatty acid
