# Supplementary figures and images for: Unsaturated Fatty Acids Affect Quorum Sensing Communication System and Inhibit Motility and Biofilm Formation of Acinetobacter baumannii
Source: Int J Mol Sci. 2018 Jan 10;19(1):214. doi: 10.3390/ijms19010214 (PMC5796163; doi:10.3390/ijms19010214)

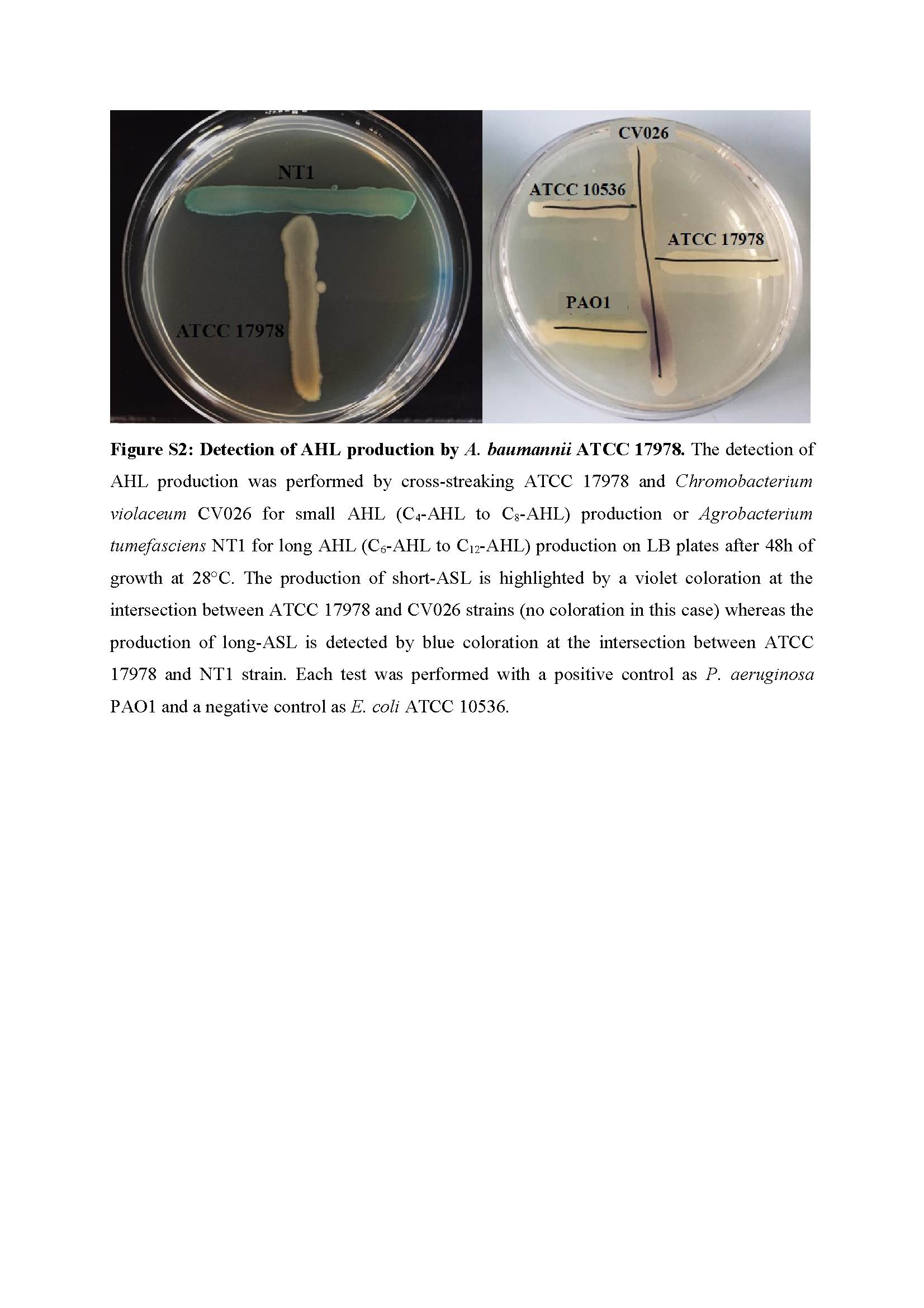

Supplement: Supplementary file 1 [file ijms-19-00214-s001.zip › Figure_S2.tiff]

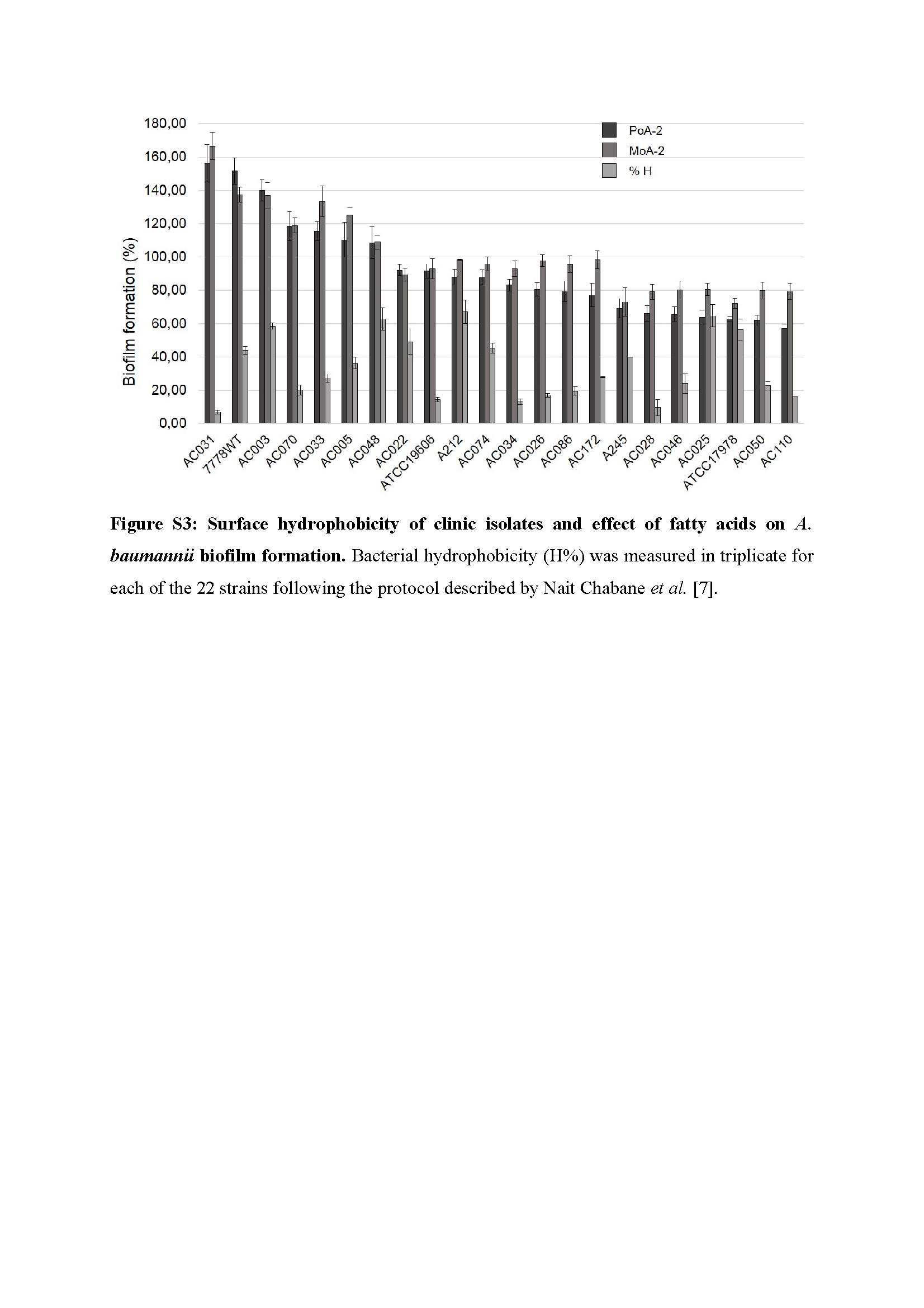

Supplement: Supplementary file 1 [file ijms-19-00214-s001.zip › Figure_S3.tiff]

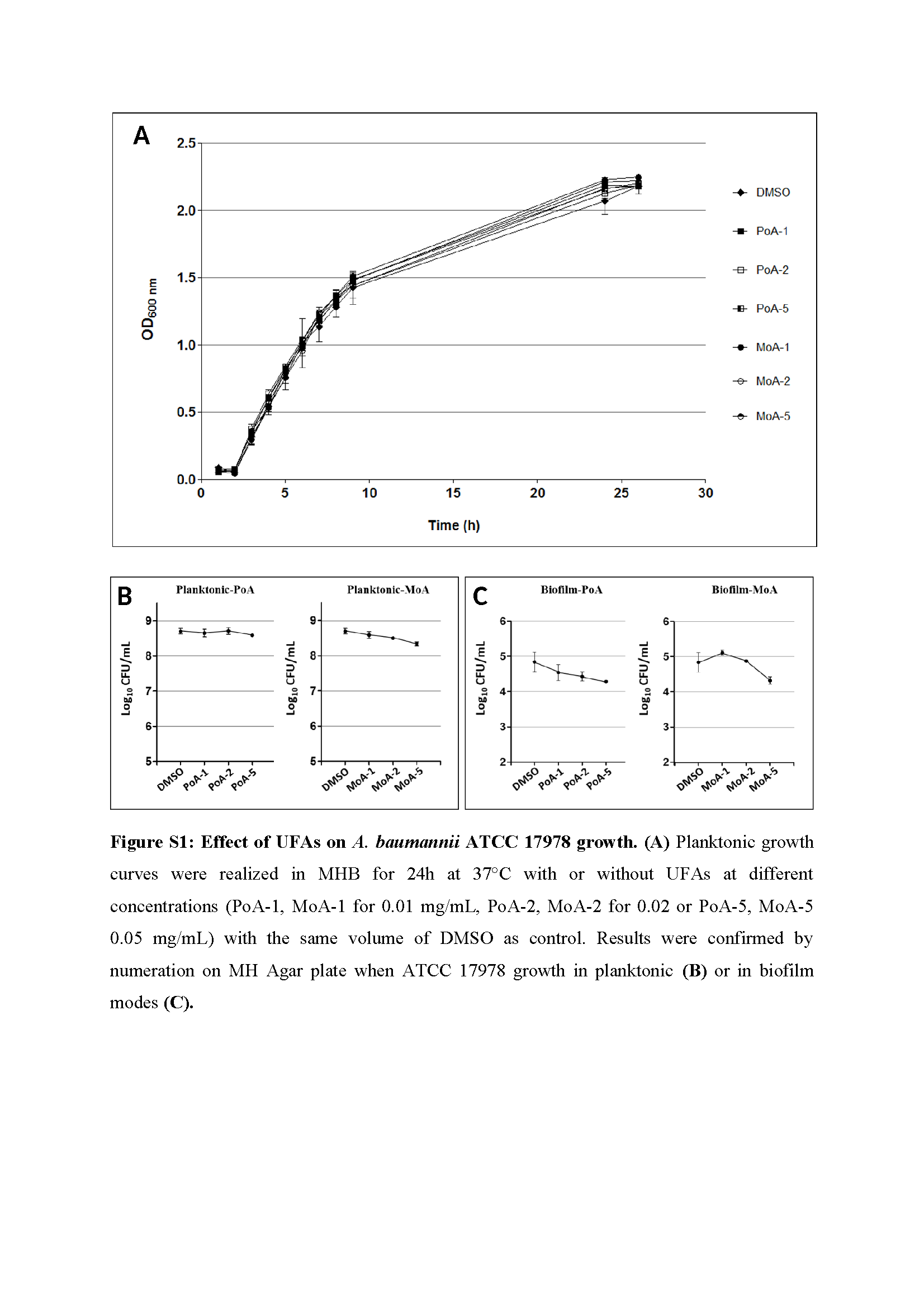

Supplement: Supplementary file 1 [file ijms-19-00214-s001.zip › Figure_S1_bis.tiff]

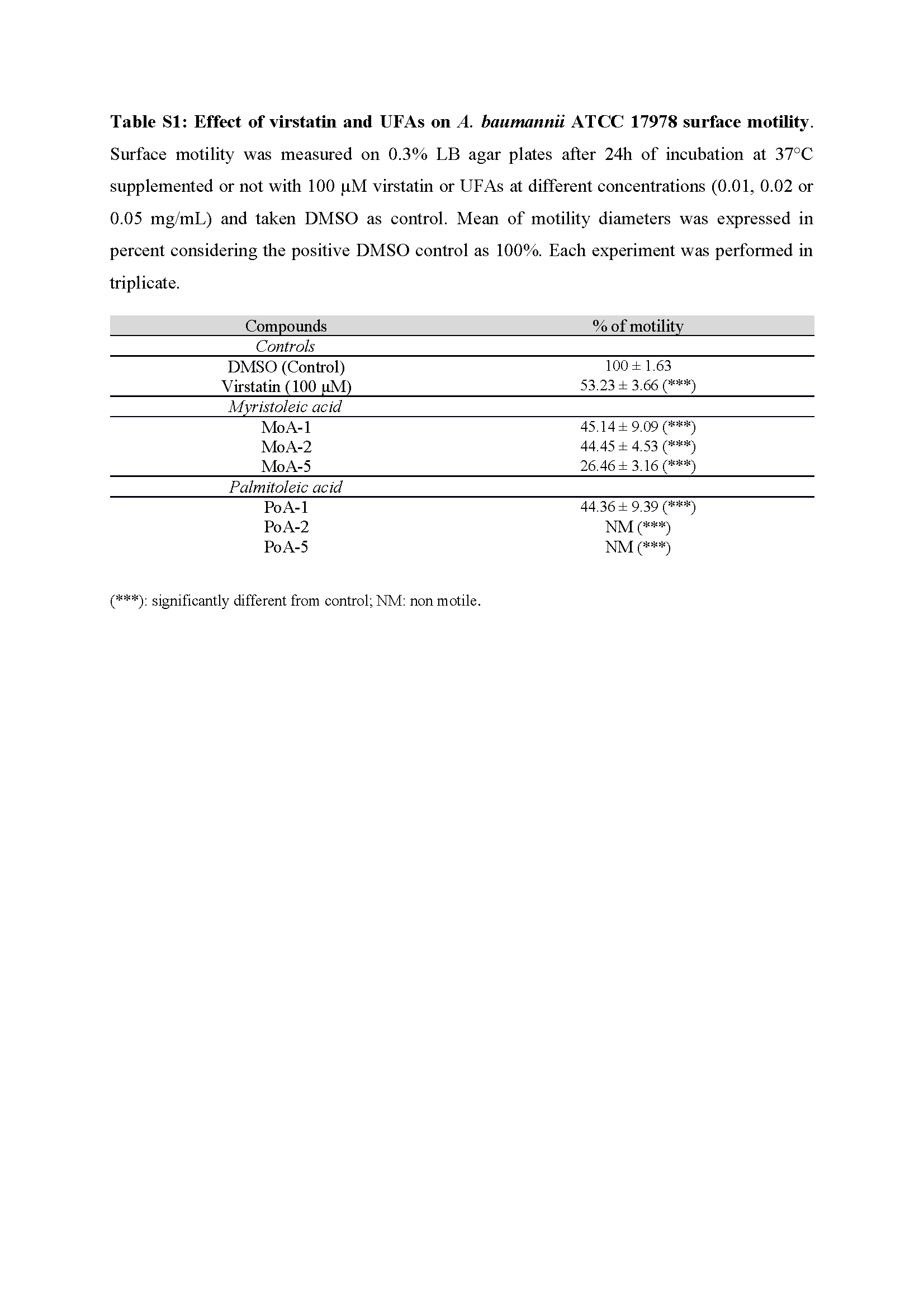

Supplement: Supplementary file 1 [file ijms-19-00214-s001.zip › Table_S1_bis.tiff]

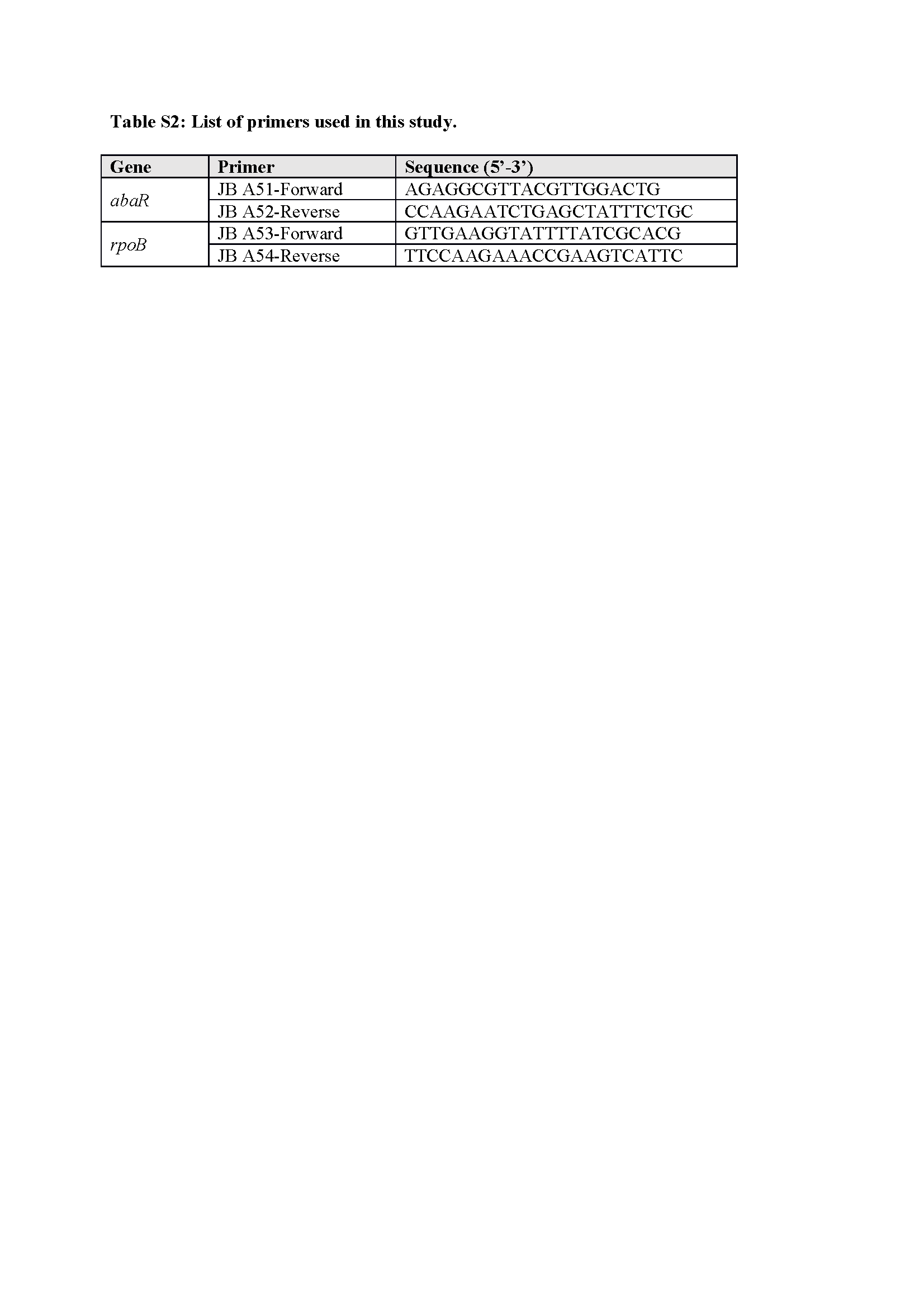

Supplement: Supplementary file 1 [file ijms-19-00214-s001.zip › Table_S2_bis.tiff]
